# Recognition of Multiple Hybrid Insulin Peptides by a Single Highly Diabetogenic T-Cell Receptor

**DOI:** 10.3389/fimmu.2021.737428

**Published:** 2021-08-30

**Authors:** Daniel Parras, Patricia Solé, Thomas Delong, Pere Santamaría, Pau Serra

**Affiliations:** ^1^Institut D’Investigacions Biomèdiques August Pi i Sunyer, Barcelona, Spain; ^2^Skaggs School of Pharmacy and Pharmaceutical Sciences (SSPPS), Department of Pharmaceutical Sciences, University of Colorado, Aurora, CO, United States; ^3^Julia McFarlane Diabetes Research Centre (JMDRC) and Department of Microbiology, Immunology and Infectious Diseases, Snyder Institute for Chronic Diseases, Cumming School of Medicine, University of Calgary, Calgary, AB, Canada

**Keywords:** autoimmunity, type 1 diabetes, epitope discovery, TCR-transgenic NOD mice, hybrid insulin peptides, antigenic promiscuity, major histocompatibility complex, genetic susceptibility and resistance

## Abstract

The mechanisms underlying the major histocompatibility complex class II (MHCII) type 1 diabetes (T1D) association remain incompletely understood. We have previously shown that thymocytes expressing the highly diabetogenic, I-A^g7^-restricted 4.1-T-cell receptor (TCR) are MHCII-promiscuous, and that, in MHCII-heterozygous mice, they sequentially undergo positive and negative selection/Treg deviation by recognizing pro- and anti-diabetogenic MHCII molecules on cortical thymic epithelial cells and medullary hematopoietic antigen-presenting cells (APCs), respectively. Here, we use a novel autoantigen discovery approach to define the antigenic specificity of this TCR in the context of I-A^g7^. This was done by screening the ability of random epitope–GS linker–I-${\text{A}}_{\beta }^{\text{g}7}$chain fusion pools to form agonistic peptide–MHCII complexes on the surface of I-${\text{A}}_{\alpha }^{\text{d}}$ chain-transgenic artificial APCs. Pool deconvolution, I-A^g7^-binding register-fixing, TCR contact residue mapping, and alanine scanning mutagenesis resulted in the identification of a 4.1-TCR recognition motif XL(G/A)XEXE(D/E)X that was shared by seven agonistic hybrid insulin peptides (HIPs) resulting from the fusion of several different chromogranin A and/or insulin C fragments, including post-translationally modified variants. These data validate a novel, highly sensitive MHCII-restricted epitope discovery approach for orphan TCRs and suggest thymic selection of autoantigen-promiscuous TCRs as a mechanism for the murine T1D–I-A^g7^-association.

## Introduction

Compelling experimental evidence have shown that type 1 diabetes (T1D) onset and progression require the activation and recruitment of autoreactive CD4+ T cells. In turn, these T cells orchestrate the activation of downstream effectors of diabetogenic autoimmunity, such as B cells and beta cell-cytotoxic CD8+ T cells (1). In non-autoimmune-prone individuals, autoreactive T cells are either deleted (thymocyte negative selection) or programmed to become autoreactive regulatory T cells (2). Both pathways require the recognition of cognate autoantigenic epitopes that are either expressed in thymic medullary epithelial cells or are delivered to the thymus by bone marrow-derived antigen-presenting cells (APCs), such as dendritic cells (DCs). As a result, thymocytes expressing TCRs capable of recognizing peptide–major histocompatibility complexes (pMHC) displaying epitopes that are selectively expressed in peripheral tissues, such as post-translationally modified epitopes or hybrid insulin peptides (HIPs), have the highest likelihood to contribute to the initiation and/or progression of autoimmunity when appropriately recruited and activated (3–5).

It has been established that MHCII polymorphisms, particularly around β-chain position 57 (β57), afford susceptibility/resistance to autoimmune diseases, including T1D, in both mice and humans, through poorly understood mechanisms (2, 6, 7). Human T1D is primarily associated with HLA-DQ polymorphisms. Whereas alleles encoding DQβ chains carrying Ala, Val, or Ser at position 57 afford risk, those carrying Asp at this position afford protection (2, 6, 7). The nonobese diabetic (NOD) mouse is homozygous for an H-2 haplotype that encodes a unique MHCII molecule, I-A^g7^ (I-Aα^d^/I-Aβ^g7^) in which the anti-diabetogenic Asp found at β57 in other murine MHCII molecules is replaced by a pro-diabetogenic Ser (2).

We have previously shown that transgenic NOD and NOD.*Rag2^–/–^* mice expressing the I-A^g7^-restricted 4.1-TCR spontaneously develop an accelerated form of T1D that results in complete destruction of pancreatic beta cells within a few weeks after birth (8). In H-2 heterozygous NOD mice expressing both pro- (I-A^g7^) and anti-diabetogenic MHC class II molecules (H2^g7/b^, H2^g7/k^, H2^g7/q^, and H2^g7/nb1^ haplotypes and transgenic I-Eα^k^, I-Aβ^d^, or I-Aβ^g7PD^ molecules), this TCR sequentially undergoes positive and negative selection/Treg cell re-programming by recognizing the pro-diabetogenic I-A^g7^ molecule on cortical thymic epithelial cells and anti-diabetogenic MHC class II molecules on hematopoietic APCs, respectively, in a β56–67-regulated manner (2, 9–13). Furthermore, selective expression of the anti-diabetogenic I-A^b^ and I-Eα^d^ molecules on DCs suppressed diabetes development in non-TCR-transgenic NOD mice, by promoting the formation of Treg cells with superior anti-diabetogenic properties than those arising in wild-type NOD mice (12, 13). Collectively, these observations exposed a potential mechanism for the MHC class II-associated susceptibility and resistance to T1D, whereby I-A^g7^ would promote the selection of MHCII-promiscuous, 4.1-like autoreactive T-cell clonotypes, capable of triggering diabetogenesis only in the absence, but not presence of anti-diabetogenic MHC class II molecules, such as I-A^b^ in mice and DQ6 in humans. That is, anti-diabetogenic MHCII would harness the intrinsic, I-A^g7^-shaped MHCII promiscuity of these TCRs to generate disease-suppressing autoregulatory T cells (2).

In an attempt to further understand the mechanistic underpinnings of the above phenomena, we sought to identify the peripheral autoantigenic target of the 4.1-TCR in the context of I-A^g7^. By screening a broad repertoire of known autoantigens and a degenerate epitope-I-A^g7^ fusion library in artificial APCs with a 4.1-TCR-transgenic reporter T-cell line, we have identified an agonistic amino acid sequence motif that, in the context of I-A^g7^, triggers robust 4.1-TCR signaling. Remarkably, we find that the 4.1-TCR recognizes seven different I-A^g7^-binding HIPs that share this motif but result from the fusion of several different chromogranin A and/or insulin C fragments, including post-translational modifications. These data suggest that 4.1-like MHCII promiscuous TCRs are also antigenically promiscuous, thus suggesting that I-A^g7^ may contribute to T1D susceptibility by expanding the antigenic repertoire of selected TCRs.

## Materials and Methods

### Mice

NOD/ShiLtJ mice were from the Jackson Lab (Bar Harbor, ME, USA). 4.1-NOD and 4.1-NOD.*Rag2* ^–/–^ mice have been described (8). All mice were bred and maintained in a specific-pathogen free (SPF) animal facility, and the experimental procedures were approved by the Ethics Committees for Animal Experimentation from the University of Barcelona and University of Calgary.

### Cell Culture

HEK-293T and JurMA cells were cultured with high-glucose Dulbecco’s modified eagle medium (DMEM) (Sigma) supplemented with 10% fetal bovine serum (FBS) (Sigma), 2 mM L-glutamine (Cultek), 1 mM sodium pyruvate (Sigma), 1% penicillin/streptomycin (Sigma) and 0.05 mg/ml gentamycin sulfate (Sigma). CHO-S cells were grown in PowerCHO-2 media (Lonza) supplemented with 8 mM glutamine and 0.05 mg/ml gentamicin sulfate. *E. coli* cultures were grown in 100 μg/ml ampicillin (Sigma)–Luria Broth (Conda).

### pMHCII Tetramers

CHO-S cells expressing peptide–I-${\text{A}}_{\beta }^{\text{g}7}$–Fc-hole/GFP and I-${\text{A}}_{\alpha }^{\text{d}}$–Fc-knob/CFP were grown in a fed-batch culture (up to 1 L) in a shaking incubator (37°C and 8% CO_2_) for 14 days with a temperature shift to 34°C when cell density reached 5 × 10^6^ cells/ml. At day 14 or when cell viability dropped below 60%, cell culture supernatants were harvested and secreted pMHCII heterodimers purified on a protein G affinity column, as previously described (14).

pMHCII monomers (50 μM) were biotinylated overnight with 10 mM biotin-protein ligase (Avidity) in 50 mM bicine buffer (pH 8.3) with 10 mM magnesium acetate, 10 mM ATP, and 5 μM d-biotin. Biotinylation reactions were buffer exchanged with a PD-10 desalting column (GE Healthcare) followed by purification of biotinylated heterodimers with a Monomeric Avidin Kit (Pierce). Biotinylated fractions were dialyzed against 20 mM Tris-HCl buffer pH 8.0 by ultrafiltration using Amicon Ultra-15 (Millipore). Biotinylated heterodimers were tetramerized at room temperature with Streptavidin-Phycoerythrin (PE) (Life Technologies) at a 4:1 molar ratio.

### pMHCII Tetramer Staining and Flow Cytometry

JurMA cells and 4.1-NOD.*Rag2* ^–/–^ splenocytes (after red blood cell lysis) were stained with 400 nM of PE-labeled pMHCII tetramers for 1 h at 37°C in 2% FBS-PBS. Splenocytes were further stained for 20 min at RT with an antibody mix containing Pacific Blue-labeled anti-CD4 (BioLegend), FITC-labeled anti-CD8 (eBioscience), and APC-labeled anti-B220 (BD) (all at 1:100 dilution). The cells were washed twice and analyzed on a Fortessa 5L Flow cytometer.

### Artificial Antigen-Presenting and TCR-Expressing JurMA Cell Lines

The HEK-293T-I-A^g7^ cell line was generated by sequential transduction of HEK-293T (ATCC, #CRL-11268) cells with lentivirus encoding for I-${\text{A}}_{\alpha }^{\text{d}}/CFP$, I-${\text{A}}_{\beta }^{\text{g}7}/GFP$, HLA-DM_α/β_/GFP, and human CD74 (invariant chain -Ii-) fused to human CD80 (hCD80) with sequential flow cytometry-based cell-sorting guided by CFP fluorescence (reporting I-${\text{A}}_{\alpha }^{\text{d}}$ expression), GFP (reporting DM_α/β_ expression), anti-hCD80 (reporting Ii, expression), and anti-I-A^g7^ (reporting I-${\text{A}}_{\alpha }^{\text{d}}$/I-${\text{A}}_{\beta }^{\text{g}7}$ heterodimer expression).

4.1- and BDC2.5-JurMA cells [derived from the TCR_β_-null Jurkat cell line J.RT3-T3.5 (ATCC) and carrying a NFAT-driven luciferase reporter] were generated by transducing JurMA cells with retroviruses encoding monocistronic, P2A-tethered TCR_α-_TCR_β_ chains.

### Generation of Plasmids Encoding for β-Cell Autoantigens

Autoantigen expression in artificial APCs was driven by transgenes encoding human invariant chain (Ii_1-80_)-β-cell autoantigen-BDC2.5 mimotope (HHPIWARMDA) fusions, or, in a transgene in which insulin (INS) and islet-amyloid polypeptide (IAPP) coding sequences were fused in-frame having their natural cleavage residues mutated to alanine. These transgenes (Ii_1-80_-β-cell autoantigen-BDC2.5mi and Ii_1-80_-INS-IAPP-BDC2.5mi) were cloned into a eukaryotic expression vector, under the control of a CMV promoter (pCMV).

Plasmids encoding I-A^g7^-binding peptides eluted from the CIITA transgenic insulinoma NIT-1 β-cell line (expressing I-A^g7^ on the cell surface) (15) or Tandem HIPs (5) were produced by VectorBuilder.

### Generation of a Combinatorial Peptide-I-${\text{A}}_{\beta }^{\text{g}7}$ Library

The expression vector encoding for I-${\text{A}}_{\beta }^{\text{g}7}$ (pCMV–GS linker–I-${\text{A}}_{\beta }^{\text{g}7}$) was PCR-linearized at the N-terminal end of I-${\text{A}}_{\beta }^{\text{g}7}$ with the primers “Backbone-F” (5’-TCGACAGCGACGTGGGCGAG-3’) and “Backbone-R” (5’-TCCGAGGCTGCTAGCGACCAG-3’). Two additional primers “Insert-F” (5’-GCTAGCAGCCTCGGAGGTNNKCGANNKNNKCTANNKGTANNKNNKGAGGGAGGTGGAGGCGGCTCAGGAGGTGGAAGCGGCGGCTCTGGAGACTCCGAAAGGCATTTCG-3’) and “Insert-Rev” (5’-CCACGTCGCTGTCGAAGCGC-3’) were used to amplify a 250-bp sequence, encoding the amino acid sequence XRXXLXVXXE–GGGGGSGGGSGGS, flanked by a 15-bp long nucleotide sequence that was homologous to the desired integration site in the linearized pCMV-I-${\text{A}}_{\beta }^{\text{g}7}$ product described above. The variable amino acid positions encoded in the epitope-coding nucleotide sequence of this 250-bp fragment used codons with random nucleotides (NNK, where N = any nucleotide, K = T/G), allowing the balanced representation of the 20 possible amino acid residues while minimizing the introduction of stop codons.

PCR-amplified products were digested with *Dpn*I (NEB) to remove potential contaminating plasmid, and DNA fragments were purified by GeneElute Gel extraction kit (Sigma) following the manufacturer’s instructions. The circular plasmid library was generated *via* a seamless recombination reaction between the linearized pCMV–I-${\text{A}}_{\beta }^{\text{g}7}$ vector and the combinatorial 250-bp sequence, catalyzed by the In-fusion HD reagent (Takara) using 300 ng of vector and a 1:3 vector:insert molar ratio. After pooling three In-fusion reactions, plasmid DNA was precipitated, resuspended in ultrapure water and used to transform electrocompetent DH10B bacteria (ThermoFisher) (exponential protocol; 2 KV, 250 Ω and 25 µF). The plasmid library was then tittered and pools of 500 colony-forming units (CFU)/well grown overnight at 37°C in U-bottomed 96-well plates containing 100 μL of LB media supplemented with 100 μg/ml of ampicillin, in a bacterial shaker at 200 rpm. The bacterial library was preserved as a glycerol stock at −80°C.

### JurMA Cell Activation Assays

#### 4.1-TCR Reactivity to Known β-Cell Autoantigens

HEK-293T–I-A^g7^ cells (2.5 × 10^4^) plated in a flat-bottom 96-well plate were transfected with 100 ng/well of pCMV-Ii_1-80_-β-cell autoantigen-BDC2.5mi DNA, using jetOPTIMUS (Polyplus). After 24 h, 10^5^ 4.1- or BDC2.5-JurMA cells were added and co-cultured for 48 h followed by luciferase activity measurement.

#### 4.1-TCR Reactivity to NIT-1-Derived I-A^g7^-Binding Peptides and β-Cell Derived HIPs

HEK-293T–I-A^g7^ cells (5 × 10^4^) plated in a flat-bottom 96-well plate were transduced with 200 ng/well of CMV promoter-driven expression vectors encoding the different peptides arrayed in monocistronic tandem configuration, using jetPRIME (Polyplus). After 24 h, 10^5^ 4.1- or BDC2.5-JurMA cells were added and incubated for an additional 48 h followed by luciferase measurement.

#### 4.1-TCR Reactivity to the Combinatorial Peptide-I-${\text{A}}_{\beta }^{\text{g}7}$ Library

Plasmid DNA from each library pool was extracted using GenElute™ Plasmid Miniprep Kit (Sigma) and 300 ng used to transfect pre-seeded 2.5 × 10^4^ HEK-293T-I-${\text{A}}_{\alpha }^{\text{d}}$ cells with jetPRIME (Polyplus). After 24 h, 10^5^ 4.1-TCR JurMA cells were added and co-cultured for 24 h and used to measure luciferase activity. Pools confirmed to elicit above-threshold luciferase activity at least twice were deconvoluted by screening sub-pools of decreasing complexity (60 cfu and 8 cfu/well), followed by single-colony screening.

#### Soluble Peptide Stimulation Assay

4.1- or BDC2.5-JurMA cells (10^5^) were co-cultured with 5 × 10^5^ pre-plated HEK-293T-I-A^g7^ cells and 0.0001–10 mg/ml peptide in a flat-bottom 96-well plate for 48 h, followed by luciferase activity measurements. All peptides (purity >90%) were obtained from GenScript^®^.

#### 4.1-TCR Reactivity to Plate-Bound Peptide–I-A^g7^ Heterodimers

Soluble epitope–I-A^g7^-knob-into-hole-based heterodimers were obtained from 72 h cultures of 2.5 × 10^4^ HEK-293T cells co-transfected with 200 ng of epitope–GS linker–I-${\text{A}}_{\beta }^{\text{g}7}$-Hole and I-${\text{A}}_{\alpha }^{\text{d}}$ -Knob expression vectors using jetPRIME (Polyplus). Immulon^®^2 high-binding ELISA plates (ThermoFisher) were coated overnight at 4°C with 25 µg/ml avidin (Pierce) followed by incubation at room temperature for 1 h with 10 µg/ml of biotin-SP-AffiniPure goat anti-human IgG-Fc (Jackson Immunoresearch). After washing with PBS, cell culture supernatants collected after 72 h from the soluble pMHCII-expressing HEK-293T transfectants were added and incubated for 2 h at room temperature. After washing with PBS, 10^5^ 4.1- or BDC2.5-JurMA cells were added to each well and the cells were cultured for 24 h before measuring pMHCII-induced luciferase activity.

### Luciferase Assay

Cells were pelleted and lysed with Cell Culture Lysis Reagent (Promega) for 20 min. Next, 30 μl of lysate/well was transferred to white-opaque flat-bottom 96-well plates (Greiner). Luciferase activity in relative light units (RLUs) was determined after injection of 100 μl/well of luciferase assay reagent (Promega), with a delay of 2 s and acquisition for 10 s with a GloMax^®^ 96 Microplate Luminometer (Promega).

### Peptide Stimulation Assay of 4.1-CD4+ T Cells

Splenic CD4+ T cells from 4.1-NOD.*Rag2^–/–^* or NOD mice were purified with CD4 (LT34) micro-beads (Miltenyi) and activated for 48 h with Dynabeads™ Mouse T-Activator CD3/CD28 (Gibco) in complete T-cell medium (RPMI-1640) (Hyclone) supplemented with 50 µM 2-mercaptoethanol (Sigma), 10% FBS, 2 mM L-glutamine, 1 mM sodium pyruvate, 1% penicillin/streptomycin, and 30 IU/ml hIL-2 (R&D). After stimulation, dynabeads were removed and expansion was maintained for 6–7 days with 100 IU/ml hIL-2 in complete T-cell medium.

Expanded cells were harvested and cocultured, in a U-bottom 96-well plate, with freshly isolated and red blood cell-lysed 10^5^ NOD splenocytes and 0.0001–10 µg/ml peptide in complete T-cell medium supplemented with 30 IU/ml of hIL-2. Cell culture supernatants were collected after 48 h and IFNγ concentrations were determined by ELISA using ELISA MAX™ Deluxe Set Mouse IFN-γ (BioLegend) according to the manufacturer’s instructions. Synthetic soluble peptides (purity >80%) were obtained from GenScript^®^.

## Results

### The 4.1-TCR Does Not Recognize Known T1D-Associated Autoantigens

**Figure 1:**
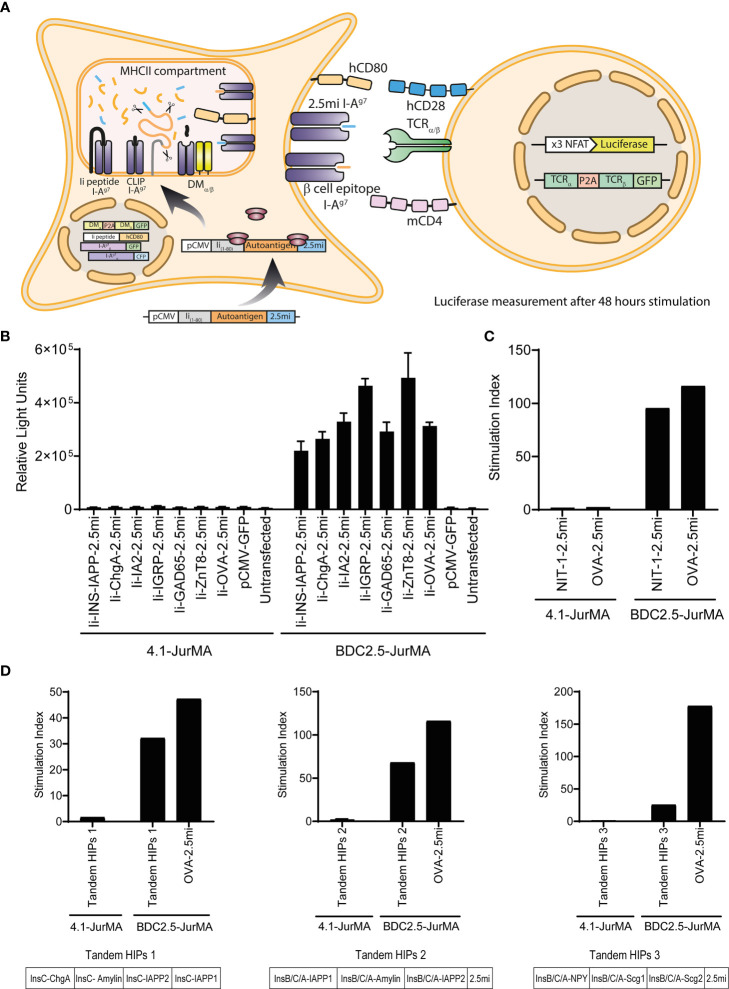
The 4.1-TCR does not recognize known T1D-associated autoantigens. **(A)** Cartoon depicting the experimental approach used to probe the agonistic properties of known T1D-associated autoantigens and peptides on 4.1-TCR-expressing cells. HEK-293T cells expressing transgenic I-${\text{A}}_{\alpha }^{\text{d}}$, I-${\text{A}}_{\beta }^{\text{g}7}$, H2-DM_α/β_, and invariant chain (CD74 or Ii) (HEK-293T-I-A^g7^) were transiently transfected with CMV promoter-driven expression vectors encoding fusions of the first 80 amino acids of murine invariant chain_1-80_ (Ii_1-80_) with specific autoantigens and a BDC2.5mi mimotope-coding sequence as an internal positive control (pCMV-Ii-autoantigen-2.5mi). These autoantigen-expressing artificial APCs were then co-cultured for 48 h with a JurMA cell-based responder T-cell line expressing mCD4, an NFAT-driven luciferase reporter, and the alpha and beta chains of the 4.1-TCR or the BDC2.5-TCR (as a positive control, recognizing the BDC2.5mi mimotope in the context of I-A^g7^), to measure luciferase activity. **(B)** The different autoantigen-2.5mi fusion-encoding plasmids readily triggered luciferase activity in BDC2.5-JurMA cells but not in 4.1-JurMA cells. **(C)** HEK-293-I-A^g7^ cells stably transfected with an expression vector encoding, in tandem, I-A^g7^-binding peptides eluted from CIITA-transgenic NIT-1 cells plus C-terminal BDC2.5mi peptide (NIT-1-2.5mi) also failed to trigger 4.1- but not BDC2.5-JurMA cell activation. **(D)** HEK-293-I-A^g7^ cells expressing potential HIPs cloned in tandem in three different expression vectors (Tandem-HIPs 1, 2 and 3, see also **Supplementary Figure S1**) triggered luciferase activity in BDC2.5-JurMA cells (reactive to its target HIP: LQTLAL-WSRMD) to levels similar to those obtained with artificial APCs transfected with a plasmid encoding an OVA-BDC2.5mi epitope fusion. The potential of the 4.1-JurMA cell line to induce luciferase expression upon TCR crosslinking was previously established with plate-bound anti-CD3 antibodies (data not shown). Stimulation indexes were calculated using the luciferase activity of every condition relative to that obtained using non-transfected artificial APCs within the same assay.

We generated an HEK-293T-based artificial APC line (16) expressing transgenic I-${\text{A}}_{\alpha }^{\text{d}}$, I-${\text{A}}_{\beta }^{\text{g}7}$, H2-DM_α/β_, and invariant chain (CD74 or Ii) to enable the processing and presentation of transiently expressed antigens in the context of I-A^g7^ (**Figure 1A**). We also generated a JurMA cell-based responder T-cell line (17) expressing mCD4, and the alpha and beta chains of the 4.1-TCR or the BDC2.5-TCR [as a positive control, recognizing the BDC2.5mi mimotope in the context of I-A^g7^ (18)]. This T-cell line carries a NFAT-driven luciferase reporter that is expressed upon TCR ligation (**Figure 1A**).

We first sought to use this approach to ascertain the ability of the 4.1-TCR to recognize previously described T1D-relevant autoantigens (19, 20), including pro-insulin (INS), chromogranin A (ChrA), islet amyloid polypeptide (IAPP), islet-specific glucose-6-phosphatase catalytic subunit-related protein (IGRP), glutamic acid decarboxylase 65 (GAD65), zinc transporter 8 (ZnT8), and islet tyrosine phosphatase 2 (I-A2). This was done by transiently transfecting the artificial APC described above with CMV promoter-driven expression vectors encoding fusions of the first 80 amino acids of murine invariant chain_1-80_ (Ii_1-80_) to each of the above autoantigens (16) (to promote antigen processing and presentation) as well as a BDC2.5mi mimotope-coding sequence at the C-terminal end as an internal positive control (pCMV-Ii-autoantigen-2.5mi) (**Figure 1A**). Whereas all of the antigen-encoding plasmids described above triggered luciferase activity in BDC2.5-JurMA cells (*via* recognition of the C-terminal BDC2.5mi epitope encoded in the various plasmids, in the context of I-A^g7^), none triggered luciferase activity in 4.1-JurMA cells (**Figure 1B**).

4.1-JurMA cells also failed to respond to artificial APCs transfected with a eukaryotic expression library (100 plasmids/pool) encoding cDNAs cloned from the murine insulinoma beta cell line NIT-1 (10^5^ cDNAs in total, including a plasmid encoding ovalbumin fused to BDC2.5mi as a positive control –pCMV-OVA-BDC2.5mi–) (data not shown). Likewise, 4.1-JurMA cells failed to respond to a single expression vector encoding Ii_1-80_ fused to a tandem sequence encoding 21 different peptides eluted from I-A^g7^ molecules expressed on the insulinoma cell line NIT-1 (**Supplementary Table S1**). These peptides were derived from Synaptotagmin 11, Teneurin Transmembrane Protein 1, Neuromodulin, Synapse-associated protein, NCAM, Secretogranin-3, Axonal Transporter of Synaptic Vesicles, Beta-site APP-Cleaving Enzyme, Synaptic Cell Adhesion Molecule, Secretogranin-2 (two peptides), Chromogranin A, NMDA 2A, Gamma-aminobutyric acid receptor-associated protein, Carboxypeptidase H, Lisch 7, Amyloid beta A4 (3 peptides), Solute Carrier Family 12 Member 7, and Reticulon 4 Receptor-Like 1 (15). As expected, the BDC-2.5-JurMA cells responded efficiently in this assay (**Figure 1C**) but not 4.1-JurMA cells.

We next considered the possibility that the 4.1-TCR might recognize a HIP. We therefore generated an expression construct encoding li_1-80_ fused to 16 previously published HIP-sequences carrying left and right arms derived from Insulin C and Chromogranin A, Amylin, IAPP, or Secretogranin 2, respectively (5). We also tested two additional expression vectors encoding 63 potential (unpublished) HIPs (**Supplementary Figure S1**). Whereas the BDC2.5-JurMA cell line responded to all these HIP-encoding constructs and the OVA-BDC2.5mi fusion positive control, 4.1-JurMA cells did not respond to any (**Figure 1D**).

### A Novel Epitope Discovery Approach

We next sought to develop and test a more systematic, sensitive, higher-throughput approach for T-cell epitope discovery, based on combinatorial epitope display, which could be used in combination with the artificial APC/TCR-transduced JurMA cell system described above.

**Figure 2:**
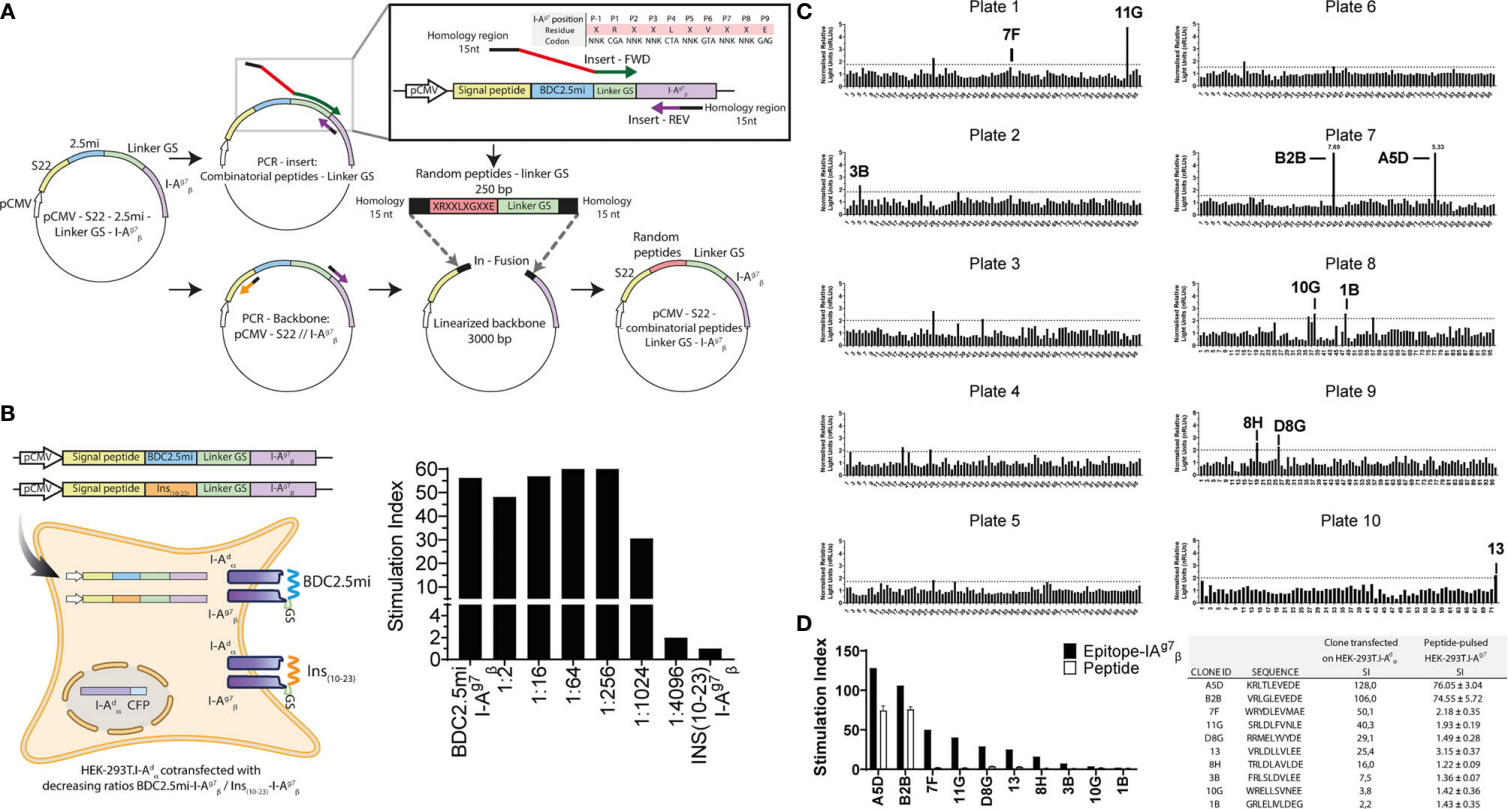
Identification of 4.1-TCR agonists with a novel epitope discovery approach. **(A)** Cartoon depicting the cloning strategy of the combinatorial peptide-I-A^g7^ library. The combinatorial peptide-coding sequences were cloned immediately upstream of the I-${\text{A}}_{\beta }^{\text{g}7}$-coding sequences into an expression vector. To build the constructs carrying combinatorial peptide-coding sequences, peptide–linker fusions carrying short arms of homology to the desired plasmid integration site were generated by PCR using degenerate primers and then cloned in-frame to the PCR-linearized expression vector upstream of I-${\text{A}}_{\beta }^{\text{g}7}$, without intervening nucleotides, using the In-fusion HD reagent. We fixed the P1, P4, P6, and P9 I-A^g7^-anchor residues (P1=R, P4=V, P6=L, and P9=E) in the peptide-coding sequence but used degenerate “NNK” codon sequences for potential TCR contact residues (P-1, P2, P3, P5, P7, and P8). **(B)** The BDC2.5mi- I-${\text{A}}_{\beta }^{\text{g}7}$ plasmid was co-transfected with increasing amounts of a similarly built Ins_10-23_- I-${\text{A}}_{\beta }^{\text{g}7}$-coding plasmid (from 1:0 to 1:4096 and 0:1 ratios, respectively) into HEK-293T cells constitutively expressing an I-${\text{A}}_{\alpha }^{\text{d}}$ transgene (HEK-293T. I-${\text{A}}_{\alpha }^{\text{d}}$) (left). The resulting cells were then used to trigger luciferase activity in BDC2.5-JurMA cells. Ratios lower than 1:1024 elicited luciferase activity (right), thus providing a sensitivity threshold for T-cell ligand screening. **(C)** Normalized relative light units (nRLUs; ratio of the RLU of each pool and the mean of RLU values of all pools tested in the same assay) induced in 4.1-JurMA cells by 96 × 10 pools of 500 clones each. RLUs higher that nRLU+3 standard deviations were considered positive. The 10 plasmid pools (out of a total of 17 positive pools identified) eliciting the highest nRLU values were deconvoluted to obtain individual agonistic mimotopes, identified in each of the plates. **(D)** HEK-293T-I-A^g7^ cells were either transfected with the agonistic plasmids from **(C)** or pulsed with the corresponding synthetic peptides (10 μg/ml) and then challenged with 4.1-JurMA cells. The panel on the left compares the stimulation indexes [ratio of luciferase units of the experimental condition *vs.* the negative control (BDC2.5mi)] obtained with plasmids *vs.* the corresponding peptides. B2B and A5D were identified as the most potent agonists. The panel on the right provides absolute values.

We cloned combinatorial peptide-coding sequences immediately upstream of a flexible 13-residue linker- and I-${\text{A}}_{\beta }^{\text{g}7}$ −coding sequences into an expression vector. The peptide–linker fusions carrying short arms of homology with the desired plasmid integration site were generated by PCR using degenerate primers and then cloned in-frame (using the In-fusion HD reagent) without intervening nucleotides immediately upstream of I-${\text{A}}_{\beta }^{\text{g}7}$ in the PCR-linearized expression vector with homologous integration sites (**Figure 2A**). We fixed the P1, P4, P6, and P9 I-A^g7^-anchor residues (P1=R, P4=V, P6=L, and P9=E) in the peptide-coding sequence, but used degenerate “NNK” codon sequences for potential TCR contact residues (P-1, P2, P3, P5, P7, and P8), where “N” can be any nucleotide and “K” is either a G or a T. By reducing codon degeneracy, we sought to maximize the likelihood that each amino acid will be represented at each open position, while minimizing the chances of introducing stop codons. These plasmids were then transfected into HEK-293T cells constitutively expressing an I-${\text{A}}_{\alpha }^{\text{d}}$ transgene (293.I-${\text{A}}_{\alpha }^{\text{d}}$). In this design, the various peptide–linker–I-${\text{A}}_{\beta }^{\text{g}7}$ protein molecules were expected to heterodimerize with the transgenic I-${\text{A}}_{\alpha }^{\text{d}}$ chain to form stable peptide-loaded MHCII complexes on the artificial APC’s surface.

We first tested the sensitivity of this approach by measuring the ability of HEK-293*T*. I-${\text{A}}_{\alpha }^{\text{d}}$ cells transfected with decreasing ratios of BDC2.5mi-I-${\text{A}}_{\beta }^{\text{g}7}$ : Ins_10-23_- I-${\text{A}}_{\beta }^{\text{g}7}$ plasmids (from 1:0 to 1:4,096 and 0:1, respectively) to activate BDC2.5-JurMA cells. As shown in **Figure 2B**, dilutions lower than 1:1,024 elicited clearly detectable responses.

### Identification of 4.1-TCR Agonists

Having defined the sensitivity threshold of this approach, and to maximize coverage without compromising sensitivity, we screened pools of 500 peptide–linker–I-${\text{A}}_{\beta }^{\text{g}7}$ clones per well distributed in ten 96-well plates, thus representing a total of 5 × 10^5^ peptides (**Figure 2C**). As cutoff luciferase activity values, we used the average of the normalized relative light units for all the screened pools plus three standard deviations. Pools eliciting responses above the cutoff value (each containing 500 clones) were deconvoluted by subcloning and re-screening until single positive clones were identified in each pool. Of the 17/960 positive pools that were identified, we deconvoluted the top 10 (**Figure 2C**). A limitation of these stringent experimental conditions is that it allows for the identification of epitopes with the highest MHC/TCR-binding affinities/avidities, potentially excluding their lower-avidity counterparts. Furthermore, although not investigated herein, it is possible that screening of bacterial pools containing fewer than 500 clones/pool might increase the sensitivity of this approach.

**Figure 3:**
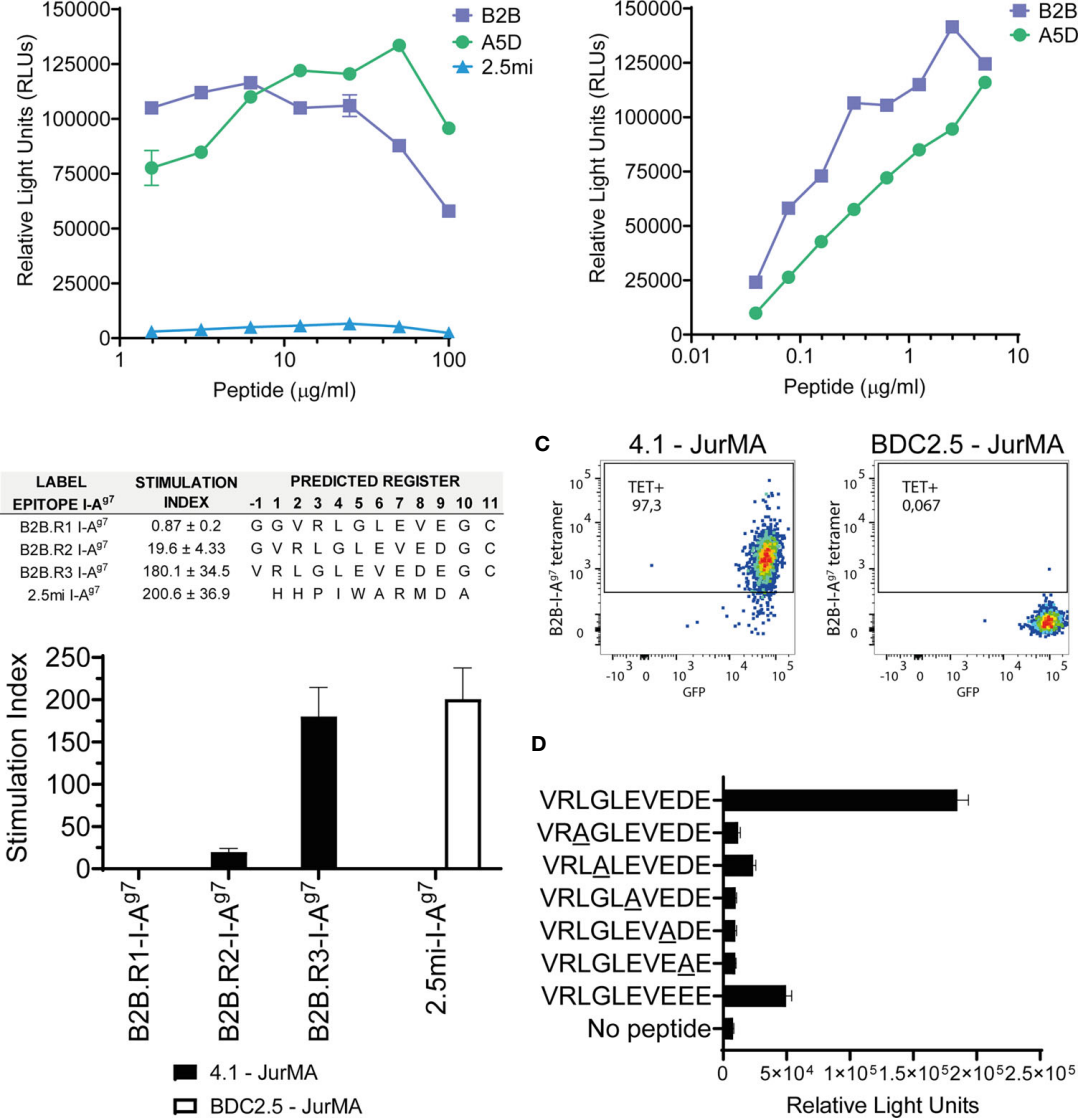
Definition of the agonistic B2B I-A^g7^-binding register. **(A)** 4.1-JurMA cells were co-cultured with HEK-293-I-A^g7^ cells and increasing concentrations of B2B, A5D, or BDC2.5mi peptides, and agonistic activity reported as RLUs. Left, 1–100 μg/ml range. Right, 0.01–10 μg/ml range. **(B)** Agonistic activity of three different plate-bound recombinant B2B-linker-I-A^g7^ pMHCIIs in which the B2B sequence was forced to bind to I-A^g7^ on three different registers *via* cys-trapping. Top, epitope sequences and absolute stimulation index values. Bottom, histogram plots. Data show that activation of 4.1-JurMA occurs preferentially *via* register 3. **(C)** Register-3 B2B-I-A^g7^ tetramers stain 4.1- but not BDC2.5-JurMA cells. GFP is a readout for TCR expression in the JurMA cells **(D)**. Synthetic B2B-like peptides carrying alanine substitutions at presumed TCR-contact residues (underlined P2, P3, P5, P7, and P8) were added (10 μg/ml) to co-cultures of HEK-293T-I-A^g7^ and 4.1-JurMA cells and luciferase activity reported in RLUs. All the alanine mutants tested had significantly lower or no agonistic activity on 4.1-JurMA cells.

We next compared the agonistic activity of the top 10 peptide–linker–I-${\text{A}}_{\beta }^{\text{g}7}$plasmids and the corresponding synthetic peptides. As expected, sensitivity was greater with the plasmids than with the peptides, as exogenously added peptides must be able to efficiently displace endogenous, HEK-293T-derived epitopes from surface I-A^g7^, to elicit cognate T-cell responses. Nevertheless, these experiments allowed the validation of two homologous peptide sequences (A5D and B2B), differing only at positions P-1 and P3, as being strong 4.1-TCR agonists (**Figure 2D**). Of these, B2B had slightly higher agonistic activity on 4.1-JurMA cells than A5D (**Figure 3A**) and was therefore chosen for further experimentation.

This approach deviates significantly from the strategy used to identify the BDC2.5mi mimotope for the BDC2.5-TCR (18, 21), which involves the screening of a peptide-I-A^g7^ library expressed on baculovirus-infected fly cells by several rounds of FACS-based sorting using soluble TCR tetramers. In the approach described herein, we combine the use of an easy to maintain, highly sensitive reporter cell line with an efficient artificial antigen presentation system that employs the highly transfectable HEK-293T cell line. These features make this screening approach more efficient, higher throughput, and less technically demanding, as expression of soluble TCRs is not a trivial undertaking.

### Definition of the Agonistic B2B I-A^g7^-Binding Register

We next sought to identify the B2B I-A^g7^ binding register(s) that most efficiently trigger(s) 4.1-TCR signaling. To do this, we used plate-bound, knob-into-hole-based B2B-linker-$\text{I}{\text{A}}_{\alpha \beta }^{\text{g}7}$ monomers secreted from transiently-transduced HEK-293T cells. As described previously, this design results in increased production yields and pMHCII structural stability than those using leucine zipper-heterodimerization domains (14, 22). Although the peptide–linker–I-${\text{A}}_{\beta }^{\text{g}7}$ library that was used to identify the B2B mimotope used four pre-defined anchor residues, it remained possible that the B2B sequence was preferentially displayed to the 4.1-TCR on alternative binding registers, such as those in which B2B uses the negatively charged Glu or Asp at P7 and P8, respectively, to anchor itself on I-A^g7^’s pocket 9. This was addressed by producing three different B2B-linker-I-A^g7^ pMHCIIs in which the B2B sequence was forced to bind to I-A^g7^ on three different registers *via* the strategic introduction of register-fixing Cysteine residues on the I-${\text{A}}_{\alpha }^{\text{g}7}$ chain and the C-terminus of B2B (cys-trap) (14, 22). We then compared the luciferase activity in 4.1-JurMA cells challenged with plate-bound monomers.

As shown in **Figure 3B**, the construct displaying B2B on register 3 (the Glu at B2B’s P9 docking into I-A^g7^’s pocket 9) had superior agonistic activity than those displaying it on registers 1 and 2 (B2B docked into I-A^g7^’s P9 pocket *via* the Glu at P7 and the Asp at P8, respectively). This was subsequently confirmed by staining 4.1-JurMA cells with pMHCII tetramers displaying B2B on register 3 (**Figure 3C**).

### Using the B2B Motif to Identify 4.1-TCR Ligands

To further validate P2, P3, P5, P7, and P8 as 4.1-TCR-contact residues in B2B anchored on I-A^g7^ *via* register 3, we compared the luciferase activity of B2B-like peptides carrying alanine substitutions at these positions. These experiments revealed strong independent contributions of each of these residues to the agonistic activity of B2B (**Figure 3D**). Of note, replacement of the Asp at P8 for a Glu reduced B2B’s agonistic activity, suggesting an important contribution for Asp at P8 on 4.1-TCR agonism.

Database searches using a degenerate amino acid sequence carrying B2B’s 4.1-TCR contact residues and permissive I-A^g7^ anchor residues did not yield naturally occurring beta cell-specific autoantigenic targets, suggesting the possibility that the 4.1-TCR might recognize a post-translationally generated and/or modified epitope, such as a HIP. We thus looked for potential HIPs sharing B2B’s 4.1-TCR contact motif XLGXEXE(D/E)X. We focused on HIPs identified in beta cell granules *via* mass spectrometry (MS) (5, 23, 24), as well as potential new HIPs in which the candidate left and right arms had been previously documented *via* MS (25). Briefly, we fused these MS-documented left and right peptides *in silico* and performed a motif search fitting the motif described above, also incorporating in the search other conserved residues for each position. The potential left and right arms included peptides derived from ChrA, Secretogranin-1 (Scg1), Secretogranin-2 (Scg2), Secretogranin-3 (Scg3), Secretogranin-5 (Scg5), IAPP, Ins1, Ins2, Pancreatic Prohormone (Ppy), Peptide YY (Pyy), Urocortin-3 (Ucn3), Neuroendocrine Convertase 1 (Pcsk1), and Neuroendocrine Convertase 2 (Pcsk2). We also considered HIPs fitting the 4.1-TCR-recognition motif upon deamidation of glutamine residues.

**Figure 4:**
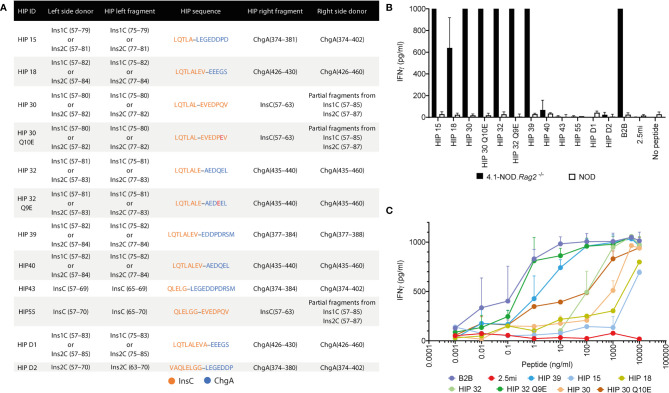
Using the B2B motif to identify 4.1-TCR ligands. **(A)** Table depicting the right and left sequence of 13 HIP candidates conforming to the B2B’s 4.1-TCR contact motif. The residue numbers on the left- and right-side donors refer to the full-length, extended polypeptide donors. The third and fifth columns indicate the residues from each donor contributing to HIP epitope formation. The fourth column provides the amino acid sequence of each HIP, with hyphens delineating the HIP junctions. **(B)** Anti-CD3/CD28 mAb-expanded splenic CD4+ T cells from 4.1-NOD.*Rag2* ^–/–^ or NOD mice were co-cultured with syngeneic NOD splenocytes as APCs in the presence or absence of the different HIPs or the BDC2.5mi mimotope as a negative control (all at 10 μg/ml). Forty-eight hours later, supernatants were harvested and the IFNγ content measured by ELISA (*n* = 3). **(C)** Responsiveness of the cells from **(B)** to the different HIPs over a broad range of peptide concentrations (0.001 ng/ml to 10 μg/ml).

Using the above criteria, we identified 13 potential HIP agonists for the 4.1-TCR (**Figure 4A** and **Supplementary Table S2**), two of which have been previously identified *via* MS (HIP 55: QLELGGEVEDPQV and HIP 30: LQTLALEVEDPQV) (25). In all these HIPs, the left arm is donated by the Insulin C-peptide, truncated at seven different carboxyterminal residues (ranging from residues 69 to 85), whereas the right arm corresponds to five different naturally occurring proteolytic products of either ChgA (*n* = 4) or InsC_57-63_ (**Figure 4A** and **Supplementary Table S2**). None of the other hybrid peptides that were probed, composed of all random combinations of *in silico* generated right and left arms from prevalent polypeptides in beta cell granules and crinosomes (including, pro-Ins1, pro-Ins2, ChrgA, IAPP, and Scg1) including variants carrying sequential truncations of one amino acid residue (a total of 7.5 million combinations), conformed to the agonistic motif described above.

Seven of these HIPs (HIPs 15, 18, 30, 30 Q10E, 32, 32 Q9E, and 39) promoted robust IFNγ secretion by anti-CD3/anti-CD28 mAb-expanded 4.1-CD4+ splenocytes (**Figure 4B**), albeit with different functional avidities (**Figure 4C**). Motif-containing HIPs D1, D2, 40, 43, and 55 lacked agonistic activity in these assays, presumably due to disruptive effects of residues at degenerate positions on 4.1-TCR engagement. Interestingly, the deamidated forms of HIPs 30 (HIP 30 Q10E) and 32 (HIP 32 Q9E) had superior agonistic activity than their wild-type counterparts (**Figure 4C**). We note that the deamidated form of the right arm of HIP 32 has been described (25). These two pro-agonistic post-translational modifications resulted in the introduction of highly favorable I-A^g7^-anchor residues for I-A^g7^’s pocket 9 (E *vs.* Q).

**Figure 5:**
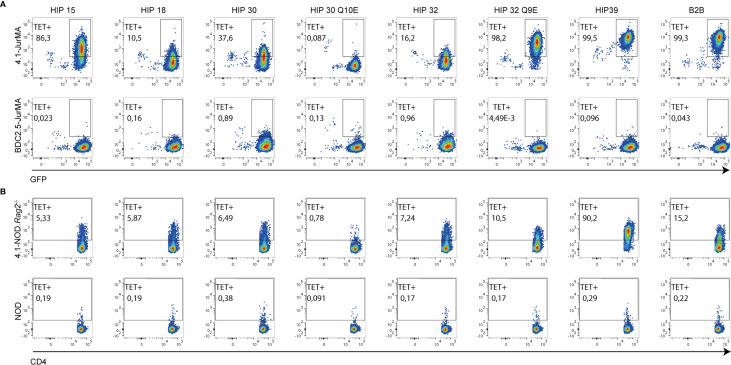
Multiple binding affinities of 4.1-TCR to different HIP-based I-A^g7^ tetramers. Representative FACS plots illustrating the ability of 7 pMHCII tetramers displaying different HIPs to bind 4.1- *vs.* BDC2.5-JurMA cells **(A)** or splenic CD4+ T cells from 4.1-NOD.*Rag2* ^−/−^ *vs.* NOD mice **(B)**. The B2B-I-A^g7^ tetramer was employed as positive control of staining. GFP is a readout for TCR expression in the JurMA cells.

Experiments using pMHCII tetramers for the seven HIPs indicated that these HIPs can be subclassified into two potential subsets with differences in functional avidity mirrored by differences in physical pMHCII-binding avidity to 4.1-JurMA cells (**Figure 5A**) and CD4+ T cells from 4.1-NOD.*Rag*2^−/−^ mice (**Figure 5B**). One of these subsets includes peptides such as HIP 39 and HIP 32 Q9E that exhibit high overall functional and physical avidity. The other subset, including the peptides HIP 15, HIP 18, HIP 30, HIP 30 Q10E, and HIP 32, is recognized with low functional and/or physical avidity. Intrinsic particularities of the different peptides within these two subgroups may account for an imperfect correlation in physical *vs.* functional avidity. For example, HIP 32 has a Q at P9, which is less favorable than a negatively charged residue at P9 (D in HIP 15). It is possible that, at high concentrations of peptide, the functional assay favors a response against HIP 32 over HIP 15, whereas the presence of D at P9 in HIP15 enhances binding to I-A^g7^, thus favoring tetramer staining. Furthermore, although HIP 30 Q10E was more potent than its wild-type counterpart HIP 30 in the IFNγ secretion assay, HIP 30 I-A^g7^ tetramers displayed higher physical binding avidity to the 4.1-TCR. This observation suggests that the deamidation Q10E in HIP 30 results in the introduction of a stronger I-A^g7^ anchor residue, shifting the register from agonistic to non-agonistic. This change would be expected to have more impact on tetramer staining (requiring a unique register binding) than in the IFNγ secretion assay where register multiplicity occurs at high concentration of soluble peptide.

## Discussion

In the work reported herein, we describe a novel autoantigen discovery approach to define the fine autoantigenic specificities of defined TCRs. We validated this approach by identifying the fine autoantigenic specificity of the 4.1-TCR, a highly diabetogenic, I-A^g7^-restricted but MHCII-promiscuous TCR whose developmental biology recapitulates MHCII allelic associations with T1D susceptibility or resistance. Previous, albeit unpublished, work using beta cell-specific cDNA expression libraries, combinatorial peptide libraries, and known recombinant autoantigens, such as insulin, phogrin, IGRP, IAPP, and ZNT8 among others, failed to yield an antigenic target for this TCR, thus precluding further dissection of the mechanisms underpinning its MHC promiscuity and diabetogenicity. Here, we used a radically different approach to address this knowledge gap. We transfected pools of cDNAs from a random epitope–GS linker–I-${\text{A}}_{\beta }^{\text{g}7}$ chain fusion library into I-${\text{A}}_{\alpha }^{\text{d}}$ chain-transgenic artificial APCs and screened the transfectants for recognition by a 4.1-TCR-transgenic T-cell line carrying an NFAT-driven luciferase reporter. Pool deconvolution, I-A^g7^-binding register-fixing, TCR contact residue mapping, and alanine scanning mutagenesis resulted in the identification of a 4.1-TCR recognition motif [XL(G/A)XEXE(D/E)X] that was shared by seven agonistic HIPs. These HIPs comprised fusions of Insulin C, truncated at seven different carboxyterminal residues, with five different naturally occurring proteolytic products of either ChgA or InsC_57-63_, including post-translationally modified variants. Collectively, our data validate a highly sensitive MHCII-restricted epitope discovery approach and demonstrate that the 4.1-TCR is also autoantigen-promiscuous, suggesting that I-A^g7^ may contribute to diabetogenesis by “expanding” the antigenic repertoire of at least some selected TCRs.

Studies on the developmental biology of the TCR cloned from the NOD islet-derived CD4+ T-cell clone NY4.1, in TCR-transgenic NOD mice and H-2-congenic or I-A- and I-E-transgenic NOD mice, revealed several unique features. First, transgenic expression of the 4.1-TCR could rapidly accelerate diabetes in both RAG-competent and RAG-deficient NOD mice (unlike the BDC2.5-TCR) (8). Second, when expressed in NOD mice carrying both pro- and anti-diabetogenic MHCII molecules, this TCR underwent negative selection or Treg cell re-programming upon recognizing unknown peptides in the context of anti-diabetogenic MHC class II molecules on hematopoietic APCs (2, 9–13). Although 4.1-NOD and 4.1-NOD.*Rag2* ^−/−^ mice spontaneously develop a highly accelerated form of diabetes, the bulk 4.1-CD4+ population of these animals is hypo-responsive to TCR stimulation as compared to bulk CD4+ T cells from NOD mice. We believe that this “anergic” state is induced by peripheral antigenic cross-reactivity to other, perhaps more abundant mimics of the HIPs, a subject that we are currently investigating.

Unlike I-A^g7^, 4.1-TCR tolerogenic MHCII molecules could not promote positive selection of 4.1-CD4+CD8+ thymocytes when selectively expressed on cortical thymic epithelial cells, indicating that positive and negative selection of the 4.1-TCR are driven by completely different peptide–MHCII complexes (pro- and anti-diabetogenic, respectively), exposed to 4.1-thymocytes by different thymic cell types (cortical thymic epithelial cells and hematopoietic APCs, respectively) at different stages of thymocyte development (2, 9–13). Studies in I-A transgenic mice carrying chimeric I-A alleles confirmed that the tolerogenic effects of protective MHCII on 4.1-TCR-induced diabetogenesis mapped to residues 56–67 of the I-Aβ chain (2, 12, 13). These observations exposed a potential mechanism for the MHC class II-associated susceptibility and resistance to T1D, whereby anti-diabetogenic MHC class II alleles (e.g., I-A^b^ in mice and DQ6 in humans) would exploit the MHCII promiscuity of disease-initiating, 4.1-like CD4+ T-cell specificities to abort the pathogenic activity of their pro-diabetogenic counterparts (e.g., I-A^g7^ in mice and DQ2/DQ8 in humans). This hypothesis implied that CD4+ T-cell specificities contributing to the initiation and/or amplification of diabetogenesis might be inherently MHCII-promiscuous and therefore capable of promoting/enhancing disease only in the absence, but not presence of anti-diabetogenic MHC class II molecules (2).

Among the HIPs tested in this study, HIP 30 shares the same contributing left-peptide fragment with HIPs recognized by the diabetes-triggering T-cell clones BDC-2.5 and BDC-6.9. This fragment is either linked to the N-terminus of insulin C-peptide (HIP 30), a ChgA peptide (BDC-2.5), or an IAPP-peptide (BDC-6.9). The presence of all three HIPs has been confidently verified through mass spectrometric analyses of NOD islets (5, 23). It was previously shown that this C-peptide fragment is the dominant C-peptide cleavage product that is co-secreted with insulin from perfused rat pancreas in a glucose-sensitive manner, implying a specific proteolytic reaction within insulin granules that leads to the generation of this cleavage product (24). A proteolytic transpeptidation reaction at this C-peptide location may thus facilitate the formation of these and other disease-relevant HIPs in beta cells.

Our finding that the 4.1-TCR is not only promiscuous for MHCII (2, 9–13) but also promiscuous for a broad repertoire of I-A^g7^-binding HIPs that cannot be presented to the 4.1-TCR by anti-diabetogenic MHCII molecules such as I-A^b^ (our unpublished data) provide fresh new insights into the potential mechanistic underpinnings of the positive and negative T1D-MHCII associations. HIPs result from the fusion of pro-insulin and chromogranin A, pro-insulin, or IAPP in beta cell secretory granules and/or crinosomes (5, 23). The low water content and high concentration of donor polypeptides in these compartments afford an optimal environment for HIP formation that cannot be replicated in the thymic cell types responsible for central tolerance. As a result, HIPs have emerged as natural ligands of autoreactive CD4+ T cells in both NOD mice (5, 26) and humans (27, 28). In turn, this implies that the peptide(s) responsible for central tolerance of 4.1-like thymocytes in the context of anti-diabetogenic MHCII on hematopoietic APCs must be different, as suggested by the inability of the HIPs described herein to trigger 4.1-TCR signaling in the context of I-A^b^. Ongoing research seeks to identify 4.1-TCR agonists in the context of I-A^d^ and I-A^b^, to ascertain the nature of the tolerogenic MHCII complexes responsible for T1D protection. Whatever the nature of these epitopes, this ability of a single TCR to engage different peptide and MHCII combinations suggest that the docking geometry of this TCR onto different pMHCII complexes may be plastic. Resolution of the x-ray crystal structures of these different complexes should provide evidence for or against this possibility.

The antigenic promiscuity of the 4.1-like TCRs may be a feature enabled by the pro-diabetogenic I-A^g7^ molecule, but “forbidden” for TCRs restricted by Asp-β57+ anti-diabetogenic MHCII molecules. Thus, it is possible that the unique structural features of I-A^g7^ (and DQ2/DQ8) enable this. It is possible that the low-avidity HIPs may not be adequately recognized by naïve 4.1-like CD4+ T cells and perhaps even antagonize T-cell activation by their high avidity counterparts or promote Treg deviation when presented by tolerogenic, immature DCs. Furthermore, it is reasonable to suspect that 4.1-like CD4+ T-cell activation (from naïve to effector) involves a multistep succession of activation events sustained by different HIPs, whereby productive recognition of the low-avidity HIPs might require pre-activation of the T cells by their high-avidity counterparts. An interesting consequence of this model is that changes in the composition of the agonist and partial agonist HIP pool would impact the effector/regulatory balance of the 4.1-like CD4+ T-cell subset. Whether or not HIP composition, including presence of deamidated versions of HIPs that promote antigenicity, change with age remains to be determined but is also a possibility.

Finally, it is possible that other HIP-reactive, I-A^g7^-restricted TCR specificities may also recognize multiple HIPs, including some of the new HIPs described herein. Although the left and right arms of the HIPs described herein have been identified *via* MS (including the full sequence of HIP 30), assessment of their pathophysiological significance (i.e., whether they are processed and presented *in vivo*) will require confirmation of their expression in islet cells or associated APCs. This will be intimately linked to the refinement and development of new HIP identification methods from complex samples, including insulin secretory granules and crinosomes. Current MS methods may not be sensitive enough to identify disease-relevant HIPs existing at low concentrations. This may account for the detection of a fairly high number of described proteolytic beta cell products in these HIP-forming beta cell structures that have not yet been found in HIPs. In this regard, our epitope identification method will help facilitate the evaluation of MS data and other proteomic techniques for the presence of the candidate epitopes.

## Data Availability Statement

The raw data supporting the conclusions of this article will be made available by the authors, without undue reservation.

## Ethics Statement

The animal study was reviewed and approved by University of Calgary and University of Barcelona Animal Care Committees.

## Author Contributions

DP executed the experimental work., PSo generated expression vectors encoding I-A^g7^, BDC2.5-TCR, and 4.1-TCR. TD provided candidate HIP peptide sequences. PSe designed the antigen discovery approach. PSe and PSa coordinated and supervised the study’s execution and wrote the manuscript with DP. All authors contributed to the article and approved the submitted version.

## Funding

This work was supported by the Ministerio de Economia y Competitividad of Spain (MINECO, RTI2018-093964-B-I00), Generalitat de Catalunya (SGR and CERCA Programmes), the Canadian Institutes of Health Research (CIHR), and the ISCIII and FEDER (PIE14/00027, PI15/0797). DP and PSo were supported by predoctoral studentships from FPU (MINECO). PSe was an investigator of the Ramon y Cajal re-integration program and was supported by a JDRF Career Development Award. The JMDRC is supported by Diabetes Canada.

## Conflict of Interest

PSa is founder and stockholder of Parvus Therapeutics, Inc.

The remaining authors declare that the research was conducted in the absence of any commercial or financial relationships that could be construed as a potential conflict of interest.

## Publisher’s Note

All claims expressed in this article are solely those of the authors and do not necessarily represent those of their affiliated organizations, or those of the publisher, the editors and the reviewers. Any product that may be evaluated in this article, or claim that may be made by its manufacturer, is not guaranteed or endorsed by the publisher.
