# Supplementary material for: Recognition of Multiple Hybrid Insulin Peptides by a Single Highly Diabetogenic T-Cell Receptor
Source: Front Immunol. 2021 Aug 30;12:737428. doi: 10.3389/fimmu.2021.737428 (PMC8435627; doi:10.3389/fimmu.2021.737428)
Supplement: Supplementary file 1 [file DataSheet_1.pdf]

Supplementary Figure 1

Tandem HIPs 1

InsC - ChgA	InsC - Amylin	InsC -IAPP2	InsC -IAPP1

Right peptides

Left peptides		Chromogranin A	Amylin	IAPP2	IAPP1
		WSRMD	KCNTA	NAARD	TPVRS
Ins C-peptide	GDLQTL	GDLQTLWSRMD	GDLQTLKCNTA	GDLQTLNAARD	GDLQTLTPVRS
	DLQTLA	DLQTLAWSRMD	DLQTLAKCNTA	DLQTLANAARD	DLQTLATPVRS
	LQTLAL	LQTLALWSRMD	LQTLALKCNTA	LQTLALNAARD	LQTLALTPVRS
	QTLALE	QTLALEWSRMD	QTLALEKCNTA	QTLALENAARD	QTLALETPVRS
	TLALEV	TLALEVWSRMD	TLALEVKCNTA	TLALEVNAARD	TLALEVTPVRS
	HLVEAL	HLVEALWSRMD	HLVEALKCNTA	HLVEALNAARD	HLVEALTPVRS
	LVEALY	LVEALYWSRMD	LVEALYKCNTA	LVEALYNAARD	LVEALYTPVRS
	VEALYL	VEALYLWSRMD	VEALYLKCNTA	VEALYLNAARD	VEALYLTPVRS

Tandem HIPs 2

InsB /C/A-IAPP1	InsB /C/A-Amylin	InsB /C/A-IAPP2	2.5mi

Right peptides

Left peptides		IAPP1	Amylin	IAPP2
		TPVRSGT	KCNTATC	NAARDP
Ins B-chain	HLVEAL	HLVEALTPVRSGT	HLVEALKCNTATC	HLVEALNAARDP
	LVEALY	LVEALYTPVRSGT	LVEALYKCNTATC	LVEALYNAARDP
	LVEALYL	LVEALYLTPVRSGT	LVEALYLKCNTATC	LVEALYLNAARDP
	LVEALYLV	LVEALYLVTPVRSGT	LVEALYLVKCNTATC	LVEALYLVNAARDP
Ins2 C -pep	QLELGGG	QLELGGGTPVRSGT	QLELGGGKCNTATC	QLELGGGNAARDP
Ins C -pep	GDLQT	GDLQTTPVRSGT	GDLQTKCNTATC	GDLQTNAARDP
Ins A-chain	TSICSL	TSICSLTPVRSGT	TSICSLKCNTATC	TSICSLNAARDP
	SICSLY	SICSLYTPVRSGT	SICSLYKCNTATC	SICSLYTPVRSGT

Tandem HIPs 3

InsB /C/A-NPY	InsB /C/A-Scg1	InsB /C/A-Scg2	2.5mi

Right peptides

Left peptides		NPY	Scg1	Scg2
		SSPETL	KADEF	IPVGSL
ins B -chain	HLVEAL	HLVEALSSPETL	HLVEALKADEF	HLVEALIPVGSL
	LVEALY	LVEALYSSPETL	LVEALYKADEF	LVEALYIPVGSL
	LVEALYL	LVEALYLSSPETL	LVEALYLKADEF	LVEALYLIPVGSL
	LVEALYLV	LVEALYLVSSPETL	LVEALYLVKADEF	LVEALYLVIPVGSL
Ins 2 C -pep	QLELGGG	QLELGGGSSPETL	QLELGGGKADEF	QLELGGGIPVGSL
Ins C -pep	GDLQT	GDLQTSSPETL	GDLQTKADEF	GDLQTIPVGSL
	GDLQTL	GDLQTLSSPETL	GDLQTLKADEF	GDLQTLIPVGSL
	DLQTLA	DLQTLASSPETL	DLQTLAKADEF	DLQTLAIPVGSL
	LQTLAL	LQTLALSSPETL	LQTLALKADEF	LQTLALIPVGSL
	QTLALE	QTLALESSPETL	QTLALEKADEF	QTLALEIPVGSL
	TLALEV	TLALEVSSPETL	TLALEVKADEF	TLALEVIPVGSL
Ins A-chain	TSICSL	TSICSLSSPETL	TSICSLKADEF	TSICSLIPVGSL
	SICSLY	SICSLYSSPETL	SICSLYKADEF	SICSLYIPVGSL

Supplementary Figure 1. Expression vectors encoding for tandem sequences of HIPs. The sequence encoding for Ii_{1-80} was C-terminal fused with a tandem sequence HIPs plus the sequence of the 2.5mi epitope and cloned in a pCMV expression vector. Following this approach, we generated 3 constructs (Tandem HIPs 1, 2 and 3) that were used to determine 4.1-TCR reactivity. Notice that the Tandem-HIPs 1 does not include the C-terminal 2.5mi sequence, as the BDC2.5-TCR natural agonistic HIP sequence (encoding for LQTLALWSRMD) which is the result of combining left InsC peptide (LQTLAL) with the right ChrA peptide (WSRMD) is included in the pool of tandem peptides.

Supplementary Table 1. Peptides eluted from I-A^{g7} molecules from NIT-1 cells that were tested for 4.1-TCR activation.

I-A^{g7} eluted peptide from NIT-1	Sequence
Synaptotagmin 11 (174-191)	VVTIQEAHGLPVMDDQTQ
teneurin transmembrane protein 1(835-849)	LQTPSQQAAKSFYDR
Neuromodulin(184-201)	QPPTETAESSQAEEEKDA
Synapse associated protein(262-279)	TPPVVIKSQLKSQEDEEE
NCAM(189-204)	SAPKVAPLVDLSDT
Secretogranin III(229-244)	IPEKVTPVAAVQDGFT
Axonal Transporter of synaptic vesicles(885-898)	VAVQAISADEEAPD
Beta-siete APP-Cleaving Enzyme(109-127)	SSNFAVAGAPHSYIDTYFD
Synaptic cell adhesion molecule(203-218)	TVTSQLMLKVHKEDDG
Secretogranin II(234-248)	DVYKTNNIAYEDVVG
Secretogranin II(420-434)	APGRGMVEALPDGLS
Chromogranin A(407-423)	RPSSREDSVEARSDFEE
NMDA 2A(36-46)	IAVLLGHSHDV
Gamma-aminobutyric acid receptor-associated protein(29-45)	VPVIVEKAPKARIGDLD
Carboxipeptidase H(348-363)	KFPPEETLKSYWEDNK
Lisch 7 (491-509)	SGRPRARSVDALDDINRP
Amyloid beta A4(237-249)	KSEFPTEADLEDF
Amyloid beta A4(475-489)	NVPAVAEEIQDEVDE
Amyloid beta A4(524-539)	ETKTTVELLPVNGEFS
Solute carrier family 12 member 7 77(6-18)	TVVPVEARADGAG
Reticulon 4 receptor-like1(366-380)	RNQISKVSSGKELTE

Supplementary Table 2. Proteolytic products contributing to the generation of HIP sequences sharing the 4.1-TCR activation motif.

HIP ID	Left side donor	Left fragment HIP	Location	HIP sequence	Right fragment HIP	Right side donor	Location
HIP 15	Ins1C (57–79)<br>or<br>Ins2C (57–81)	Ins1C (75–79)<br>or<br>Ins2C (77–81)	Islet as Ins2C(61-81) (MHCII peptidome), pLN (MHCII peptidome), spleen (MHCII peptidome), DCGs, secretome, crinosomes *(23)*, beta cell extracts *(5)*	LQTLA–LEGEDDPD	ChgA(374–381)	ChgA(374–402)	DCGs, secretome, crinosomes *(23)*
HIP 18	Ins1C (57–82)<br>or<br>Ins2C (57–84)	Ins1C (75–82)<br>or<br>Ins2C (77–84)	Secretome, crinosomes *(23)*	LQTLALEV–EEEGS	ChgA(426–430)	ChgA(426–460)	crinosomes *(23)*
HIP 30	Ins1C (57–80)<br>or<br>Ins2C (57–82)	Ins1C (75–80)<br>or<br>Ins2C (77–82)	Islet (MHCII peptidome), pLN (MHCII peptidome), spleen (MHCII peptidome), DCGs, secretome, crinosomes *(23)*, beta cell extracts *(5)*, peptide forming HIP *(24)*	LQTLAL–EVEDPQV	Ins(57–63)	Partial fragments from<br>Ins1C (57–85)<br>Ins2C (57–87)	Islet (MHCII peptidome), pLN (MHCII peptidome), spleen (MHCII peptidome), DCGs, secretome, crinosomes *(23)* beta cell extracts *(5)*, peptide forming HIP *(24)*
HIP 30<br>Q10E	Ins1C (57–80)<br>or<br>Ins2C (57–82)	Ins1C (75–80)<br>or<br>Ins2C (77–82)	Islet (MHCII peptidome), pLN (MHCII peptidome), spleen (MHCII peptidome), DCGs, secretome, crinosomes *(23)*, beta cell extracts *(5)*, peptide forming HIP *(24)*	LQTLAL–EVEDPEV	Ins(57–63)	Partial fragments from<br>Ins1C (57–85)<br>Ins2C (57–87)	Partial right part post-translational modified [PqVEQ -> PeVEQ] found in pLN (MHCII peptidome) *(23)*
HIP 32	Ins1C (57–81)<br>or<br>Ins2C (57–83)	Ins1C (75–81)<br>or<br>Ins2C (77–83)	DCGs, secretome, crinosomes *(23)*	LQTLALE–AEDQEL	ChgA(435–440)	ChgA(435–460)	DCGs, crinosomes *(23)*
HIP 32<br>Q9E	Ins1C (57–81)<br>or<br>Ins2C (57–83)	Ins1C (75–81)<br>or<br>Ins2C (77–83)	DCGs, secretome, crinosomes *(23)*	LQTLALE–AEDEEL	ChgA(435–440)	ChgA(435–460)	Partial right part post-translational modified [AEDqEL -> AEDeEL] in crinosomes *(23)*
HIP 39	Ins1C (57–82)<br>or<br>Ins2C (57–84)	Ins1C (75–82)<br>or<br>Ins2C (77–84)	Secretome, crinosomes *(23)*	LQTLALEV–EDDPDRSM	ChgA(377–384)	ChgA(377–388)	crinosomes *(23)*
HIP40	Ins1C (57–82)<br>or<br>Ins2C (57–84)	Ins1C (75–82)<br>or<br>Ins2C (77–84)	Secretome, crinosomes *(23)*	LQTLALEV–AEDQEL	ChgA(435–440)	ChgA(435–460)	DCGs, crinosomes *(23)*
HIP 43	InsC (57–69)	InsC (65–69)	pLN (MHC II peptidome), DCGs, secretome, crinosomes *(23)*, beta cell extracts *(5)*	QLELG–LEGEDDPDRSM	ChgA(374–384)	ChgA(374–402)	DCGs, secretome,crinosomes *(23)*
HIP55	InsC (57–70)	InsC (65–70)	pLN (MHCII peptidome) as Ins1C (61-70) and Ins2C (61-70), DCGs, secretome, crinosomes *(23)* beta cell extracts *(5)* HIP-forming peptide as Ins2C(60-70) *(24)*	QLELGG–EVEDPQV	Ins(57–63)	Partial fragments from<br>Ins1C (57–85)<br>Ins2C (57–87)	Islet (MHCII peptidome), pLN (MHCII peptidome), spleen (MHCII peptidome), DCGs, secretome, crinosomes *(23)*, beta cell extracts *(5)*, peptide forming HIP *(24)*
HIP D1	Ins1C (57–83)<br>or<br>Ins2C (57–85)	Ins1C (75–83)<br>or<br>Ins2C (75–85)	Islet (MHCII peptidome), DCGs, secretome, crinosomes *(23)* , beta cell extracts *(5)*	LQTLALEVA–EEEGS	ChgA(426–430)	ChgA(426–460)	Crinosomes *(23)*
HIP D2	Ins2C (57–70)	Ins2C (63–70)	pLN (MHCII peptidome) as Ins1C (61-70) and Ins2C (61-70), DCGs, Secretome, Crinosomes *(23)* beta cell extracts *(5)* HIP-forming peptide as Ins2C(60-70) *(24)*	VAQLELGG–LEGEDDP	ChgA(374–380)	ChgA(374–402)	DCGs, secretome, crinosomes *(23)*

Orange: Insulin C
Blue: Chromogranin A
